# 3D nanopolymerization and damage threshold dependence on laser wavelength and pulse duration

**DOI:** 10.1515/nanoph-2022-0629

**Published:** 2023-01-13

**Authors:** Danielius Samsonas, Edvinas Skliutas, Arūnas Čiburys, Lukas Kontenis, Darius Gailevičius, Jonas Berzinš, Donatas Narbutis, Vytautas Jukna, Mikas Vengris, Saulius Juodkazis, Mangirdas Malinauskas

**Affiliations:** Laser Research Center, Physics Faculty, Vilnius University, Sauletekio Ave. 10, Vilnius, Lithuania; Light Conversion, Keramikų 2b, Vilnius, LT-10223, Lithuania; Physics Faculty, Institute of Theoretical Physics and Astronomy, Vilnius University, Sauletekio Ave. 3, Vilnius, LT-10257, Lithuania; Optical Sciences Centre and ARC Training Centre in Surface Engineering for Advanced Materials (SEAM), School of Science, Swinburne University of Technology, Hawthorn, VIC 3122, Australia; WRH Program International Research Frontiers Initiative (IRFI) Tokyo Institute of Technology, Nagatsuta-cho, Midori-ku, Yokohama 226-8503, Japan

**Keywords:** 3D printing, multi-photon phenomena, non-linear absorption, optical damage threshold, photo-polymerization, ultrashort laser pulses

## Abstract

The dependence of the polymerization and optical damage thresholds in multi-photon polymerization (MPP) lithography was studied using a broadly-tunable laser system with group delay dispersion (GDD) control. The order of non-linearity and the light–matter interaction mechanisms were investigated using the resolution bridges method for non-photosensitized SZ2080^TM^ and photosensitized SZ2080^TM^ + IRG369 prepolymers. Energy deposition, voxel dimension growth, and the size of the dynamic fabrication window (DFW) were measured in the 700–1300 nm wavelength range at three different pulse durations measured at the sample – 100, 200 and 300 fs. Polymerization was observed at all wavelengths and pulse durations without significant differences in the achieved minimal spatial dimension ( <300 nm). This was achieved despite the broad range of excitation wavelengths used which spanned two- and three-photon absorption bands, and the differences in the absorption spectra of the prepolymers. The lateral and longitudinal voxel growth dynamics revealed an abrupt change in the power dependence of polymerization and a significant variation of the DFW – from 1 at 1250 nm to 29 at 700 nm. This result can be interpreted as a consequence of a change in the instantaneous refractive index and a lowering of the polymerization but not the damage threshold. The optimization of energy delivery to the material by a wavelength-tunable laser source with pulse duration control was experimentally validated. These findings are uncovering the complexity of polymerization mechanisms and are useful in further development of MPP technology.

## 1 Introduction

Multi-photon polymerization (MPP) enables fabrication of both 3D features down to the 100 nm scale, at a resolution beyond the diffraction limit, and functional structures with dimensions ranging from tens of microns up to a few millimeters [1], [2], [3]. Current development is focused on high-throughput multi-focus nanolithography setups that approach 3D printing speeds of ten million voxels per second and are already being used for rapid prototyping [4] as well as production of 3D metamaterials [5] and arrays of microlenses [6, 7]. Some straightforward technical solutions are being introduced to reduce complexity of the MPP workstations and sample handling [8]. At the same time, novel-concept MPP techniques are still being developed, e.g., interparticle chemical bonding by exciting holes in semiconductor quantum dots with potential application in free-form quantum dot optoelectronics [9].

MPP is initiated when several photons are simultaneously absorbed by the prepolymer molecule bringing it to an excited state that ultimately forms a radical which then participates in a chain polymerization reaction, leading to chemical cross-linking. The photochemical events leading to radical formation can follow a multitude of pathways involving different molecular states and the yield of radical production may differ accordingly. In addition, two-photon (2P), three-photon (3P), and higher-order absorption coefficients may differ strongly and have different wavelength dependencies because particular selection rules apply for molecular states with different symmetries. At some wavelengths, where the contributions of different orders of non-linear absorption (*N*) are similar in magnitude, absorption order can become non-integer, which is especially noticeable at off-peak absorption wavelengths [10]. A lot of effort has been spent in synthesizing and studying photoinitiators (PI) in order to increase polymerization efficiency, spatial resolution and fabrication throughput [11]. And, on the contrary, it seems that localized polymerization can be induced without using the PI at all [12], which adds further depth to the debate on photo-excitation mechanisms.

The peak intensity, spectral bandwidth and pulse duration of ultrashort pulses are inherently intertwined, and it is not trivial to control each of these parameters separately at the sample. Failure to realize this may lead to apparent dependence of non-linear absorption on the pulse duration in several ways. Firstly, typical spectral bandwidths of ultrashort pulses used in MPP are on the order of 5–50 nm. Such spectrally broad pulses are sensitive to the group delay dispersion (GDD) of the optical train of MPP microscope and can considerably stretch in time. Different frequency components experience different delays, the pulse becomes chirped, and its peak intensity is reduced. Such an intensity decrease will reduce the efficiency of any multi-photon process including MPP. Therefore, the dispersion of the microscope optics needs to be accounted for by introducing the same amount of the opposite temporal pre-chirp for the input pulses. This is commonly achieved by chirped mirrors [13], various prism-based, or grating-based compressors [14]. For shorter pulses on the order of 50 fs, third-order dispersion (TOD) must also be considered. TOD leads to an asymmetric temporal chirp of the pulse and therefore is more difficult to compensate, requiring techniques such as temperature tuning to be employed [15]. Secondly, shorter pulses from different laser sources will cover a wider portion of non-linear absorption spectrum. If non-linear material absorption is not constant over the pulse bandwidth, it will manifest itself as an apparent dependence of the non-linear absorption coefficient on the duration of the pulse. Finally, the photo-initiation reactions may exhibit coherent effects dependent on phase relationships between different frequency components of the pulse [16], which even for pulses with identical autocorrelation traces and the bandwidths would result in different photoproduct yields.

While there are numerous advances in making MPP more versatile and accessible, for practical reasons, most research uses fixed-wavelength sources with limited dispersion control. The most popular wavelengths are: 780 nm [17], 800 nm [18] (400 nm [19]), 1030 nm [20] (515 nm [21]) and 1064 nm [22] (532 nm [23]), corresponding to the fundamental wavelength and harmonics of erbium-doped fiber, Ti-sapphire, Yb:KGW and Nd:YAG lasers, respectively. While it is natural to employ laser sources with the highest availability, it also has to be acknowledged that both the wavelength and the temporal dispersion of the pulses have been observed to significantly influence laser micro-machining results [24], [25], [26]. Fundamentally, MPP is just another process employing non-linear optical phenomena, and there is good reason to expect that it will also be sensitive to these experimental variables.

The aim of this study was to investigate the MPP dependence on wavelength and pulse duration using the resolution bridge (RB) method. Employing a newly developed wavelength-tunable laser system with pulse GDD control, we have explored how these variables affect the polymerization and optical damage thresholds, the dynamic fabrication window (DFW), and voxel growth dynamics in the 700–1300 nm wavelength range at three different pulse durations (100, 200 and 300 fs) in photosensitized and non-photosensitized SZ2080^TM^ prepolymer.

## 2 Materials and methods

### 2.1 Experimental setup

**Figure 1:**
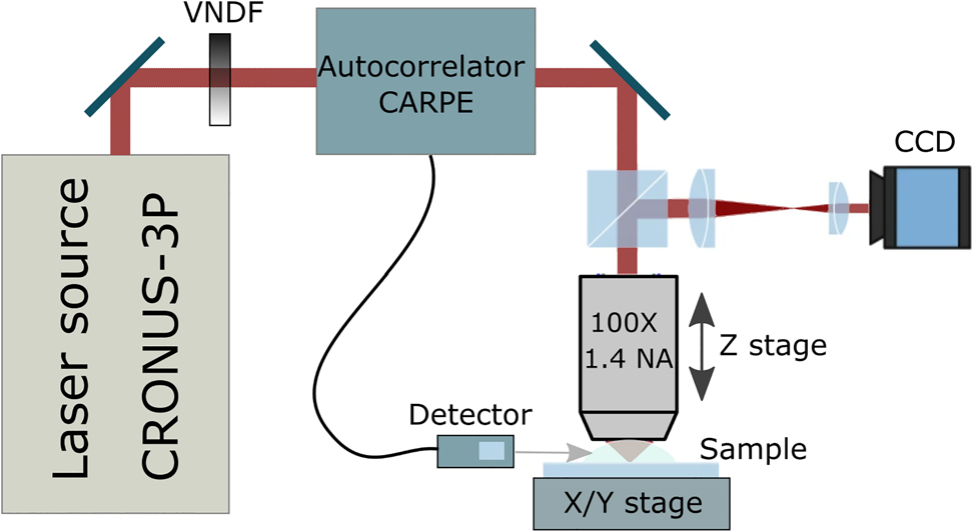
Layout of the experimental setup: wavelength-tunable laser system with pulse GDD control (CRONUS-3P), *τ* measurement (CARPE) and beam attenuation control (VNDF), custom-made microscope setup: piezo-stage stack for *X* and *Y* movement, *Z*-axis stage, focusing objective, CCD camera.

The experiment was carried out using a broadly wavelength-tunable laser source with automated pulse GDD control (CRONUS-3P, Light Conversion). An autocorrelator capable of working under a high-NA objective (CARPE, APE Angewandte Physik and Elektronik) was used to measure pulse duration at the sample. A home-built microscope with a Zeiss Plan-Apochromat 100 × 1.4 NA oil-immersion objective was used to focus the excitation light on the sample. An automated 3-lens telescope integrated into the CRONUS-3P laser system was calibrated to maintain constant beam size and collimation while tuning the wavelength. Objective lens overfill was set so that beam size at 1/*e* ^2^ was equal to objective entrance pupil. The sample was placed with the prepolymer side facing down. Sample translation was provided by a combined stage containing XY piezo-stages (P-563 PIMars, Physik Instrumente) and a motorized XY scanning stage (8MTF-75LS05, Standa). Another motorized translation stage (8MT167-100, Standa) was employed for objective positioning. Average power *P*_ave_ was controlled using an automated variable neutral density filter (VNDF) and measured before the objective using a power meter: PD300 (Ophir) for *λ* ≤ 1100 nm and 3A (Ophir) for *λ* > 1100 nm. Power loss at objective entry pupil was considered to be constant at 13.5%. During the experiment, the repetition rate was set to 1 MHz and the pulse duration after the objective was set to 100, 200 and 300 fs for each *λ*. Additional measurements were taken for several *λ* ≥ 1050 nm, where the GDD tuning range was sufficient for achieving 500 fs pulse duration. The layout of the experimental setup is depicted in Figure 1.

### 2.2 Laser system

**Figure 2:**
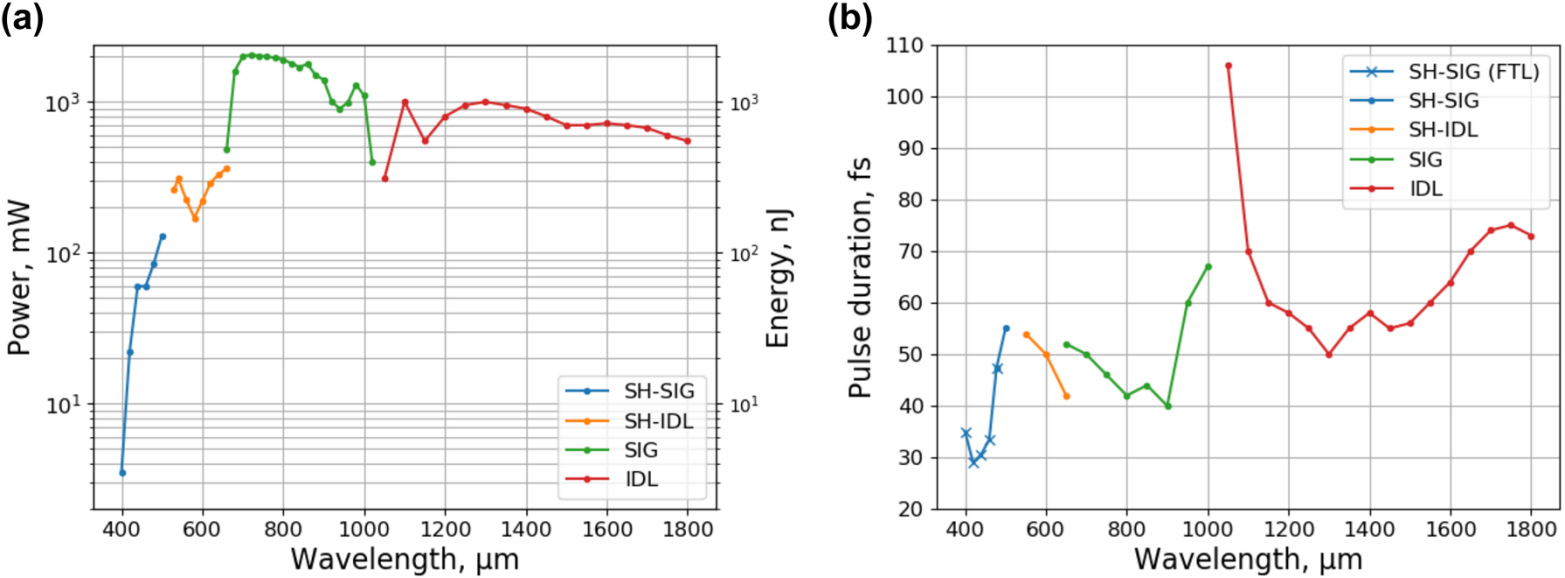
OPA parameters at system output, (a) – average power and pulse energy, and (b) – minimum pulse duration. Fourier-transform-limited values are given in the 400–480 nm range.

The laser source used in this study is a combination of an amplified Yb:KGW laser and an optical parametric amplifier (OPA) followed by automated pulse GDD and beam size control. The standard system was extended for operation in the VIS range for this study. The laser provides µJ-level pulses down to 50 fs at repetition rates of up to 2 MHz and is tunable in the range from 400 nm to 1800 nm. Figure 2(a) shows the output power of the laser system, measured with a thermopile sensor (3A, Ophir). Output above 1050 nm is an idler beam (IDL), 650–1010 nm is a signal beam (SIG), 530–650 nm is the second harmonic (SH) of the idler beam (SH-IDL), and 400–500 nm is the SH of the signal beam (SH-SIG). Minimum pulse duration at laser output was measured using a scanning autocorrelator (GECO, Light Conversion) and is presented in Figure 2(b). Autocorrelator measurements were limited to 500–2000 nm wavelength range. Therefore, Fourier-transform-limited (FTL) pulse duration is provided for the 400–480 nm range. FTL values were estimated from the OPA spectral bandwidth measurements recorded using visible-range (AvaSpec-3648, Avantes) and near-infrared (NIRQuest-512, Ocean Optics) spectrometers.

**Figure 3:**
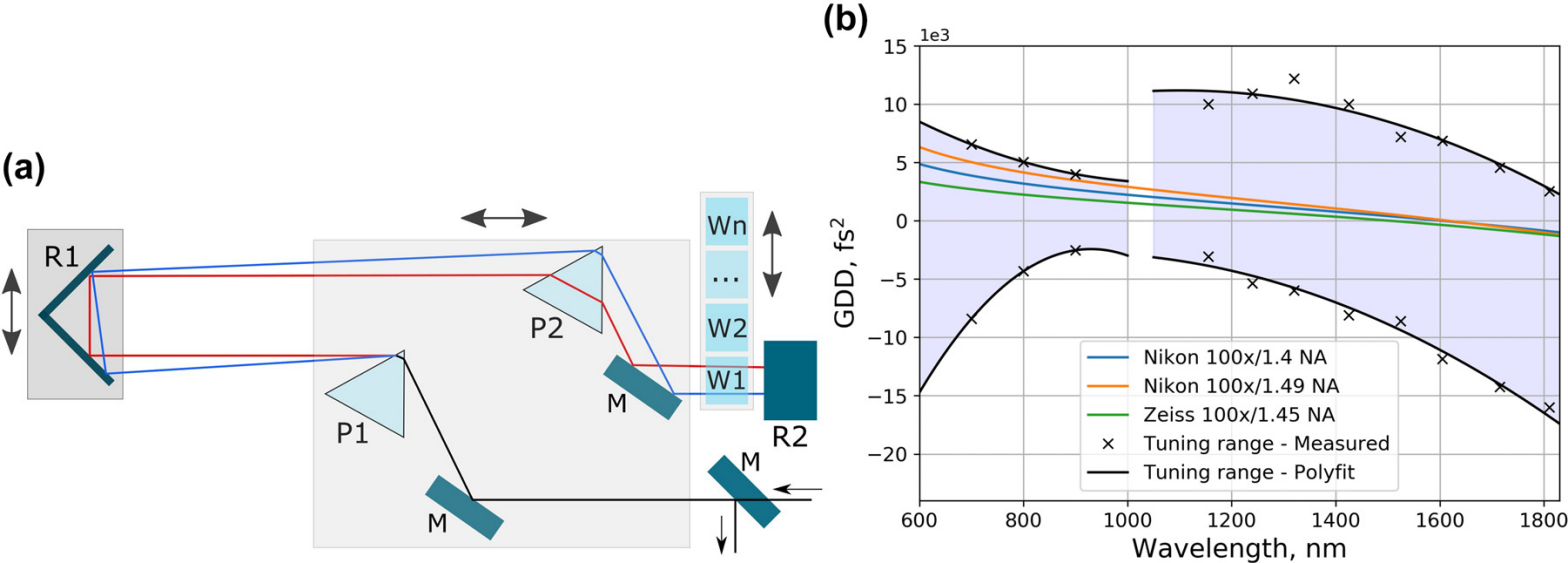
Group delay dispersion control: (a) – Principal diagram of the compressor. M – mirror, P – prism, R – retroreflector, W – glass plate. Double arrows represent compressor tuning by translating the components in the greyed-out areas. (b) – Compressor GDD tuning range compared to the dispersion of several high-NA immersive objectives.

Automated beam and pulse conditioning is essential to the investigation of wavelength and pulse duration effects as they allow to study non-linear processes in MPP as function of a single parameter, while maintaining all other laser parameters constant. The compressor unit is capable of automated pulse GDD control over the entire 400–1800 nm OPA tuning range. In order to provide positive and negative GDD compensation capability with a single set of prisms, a design utilizing a prism compressor with a set of glass plates was chosen. Figure 3(a) shows the principal diagram of the compressor. Negative chirp is induced by tuning tip-to-tip prism spacing and the amount each prism is inserted into the beam, while positive chirp is created in discrete amounts by inserting or removing of glass plates. Such a combination expands GDD pre-compensation in the NIR range where many optical glasses exhibit anomalous dispersion. Similar folded prism compressor designs have been employed before [27, 28]. However, the presented design ensures that the optical path length through the compressor and, therefore, the pulse delay changes are minimized. Over the entire wavelength range, the pulse delay varies only by a few ps when GDD is tuned by changing the prism spacing. Insertion of glass plates adds from 16 to 93 ps delay for pulses in the NIR range when a positive chirp is needed. Such small delays can be compensated using a compact delay line suitable for most microscopy setups. This aspect is important in applications employing more than one laser pulse, for example, CARS, SRS and SFG, where temporal overlap between interacting pulses must be maintained [29].

Figure 3(b) shows the measured GDD compensation range of the compressor, as well as the theoretical dispersion values required for the compensation of several high-NA objectives typical in MPP applications. The objective GDD values were estimated from the optical prescription information provided in patents for 100 × 1.4 NA [30] and 100 × 1.49 NA [31] objectives patented by Nikon, and 100 × 1.45 NA objective patented by Zeiss [32]. The objective is usually the main source of dispersion in microscopy and nanopolymerization setups, and the presented compressor design is able to compensate the objective GDD over a broad range with a margin for other optical components in the setup.

### 2.3 Polymerization threshold and voxel size measurements

**Figure 4:**
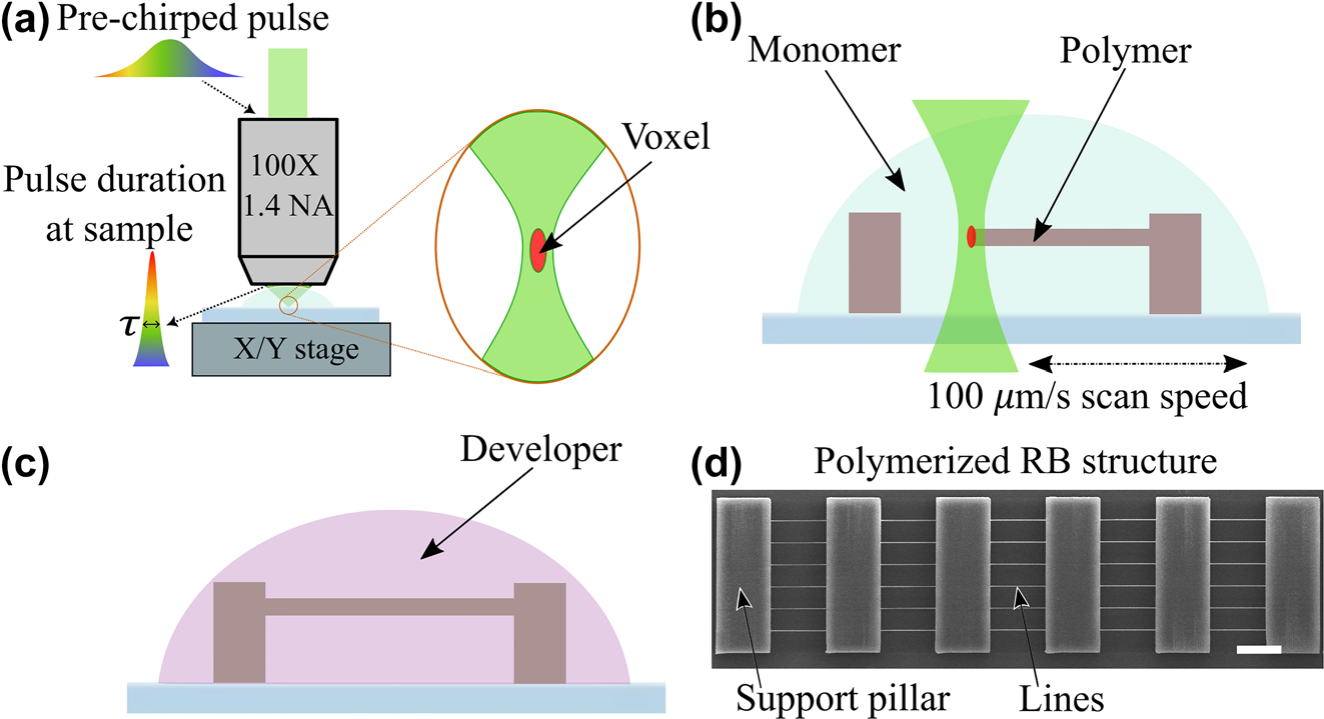
Experimental procedure: (a) – focusing of the pre-chirped pulse into the sample; (b) – multi-photon polymerization of RB structure; (c) – development of the sample; (d) – SEM image of an entire RB object with lines and support pillars. Scale bar is 20 µm.

The intensity threshold values for polymerization were determined using the resolution bridge (RB) technique [33]. This technique is based on the manufacturing of thin lines of polymer suspended between large support columns. The experimental procedure and an example of a scanning electron microscopy (SEM) image of an RB object is shown in Figure 4. Each line had a length of 75 µm and was polymerized at a fixed intensity (*I*) in a single stage movement at a typical velocity of 100 μm/s. The intensity level was increased for each adjacent line from the pre-threshold value until the onset of damage. An offset of 8 µm from the monomer-substrate interface was used. To determine the voxel size, the width and height of the polymerized lines were measured from the SEM images obtained from perpendicular and 45°-tilted views. A region-of-interest (ROI) of 1000 × 200 pixels was defined at the center of each polymerized line. It included the center ∼25 µm of the line for analysis. Then, each pixel column in the defined ROI was evaluated to determine the top and bottom boundaries of the line. The difference between the top and bottom boundaries was used to calculate the mean size value and its variation across the ROI for each polymerized line.

### 2.4 Prepolymer material

**Figure 5:**
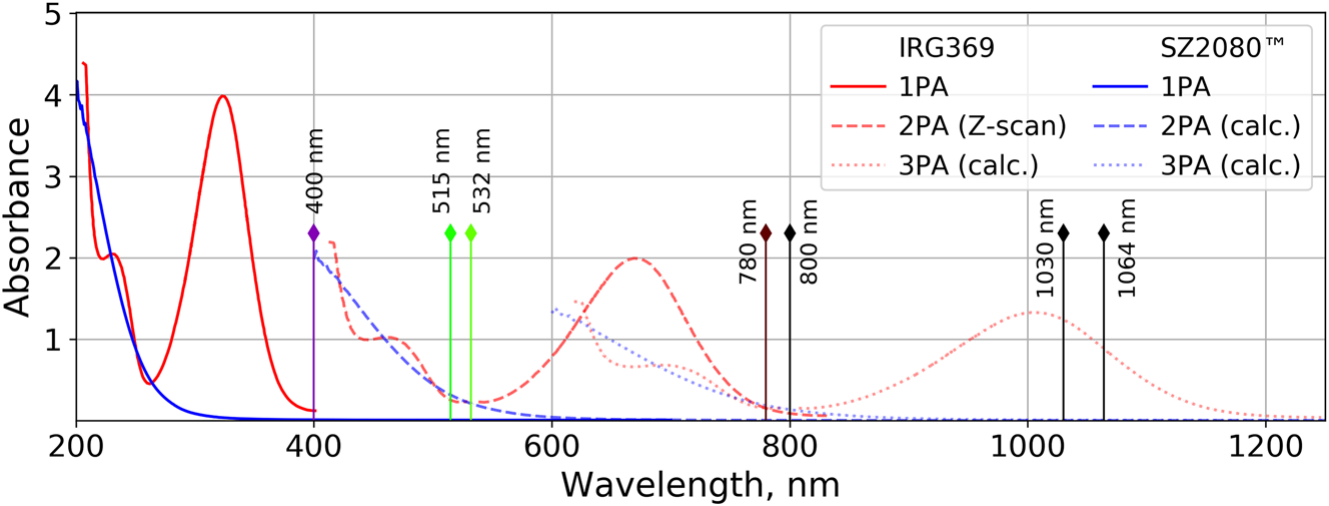
Absorbance spectra of the prepolymer and the photoinitiator. Blue lines represent measured 1P absorbance spectra (solid line) [34] and the corresponding expected 2P (dashed line) and 3P (dotted line) absorbance spectra of the SZ2080^TM^ prepolymer, marked as calc. in the legend. Red lines represent measured 1P absorbance spectra of the IRG369 photoinitiator (solid line), the same spectra shifted to the 2PA maximum measured using the Z-scan technique (dashed line) [35], and the corresponding expected 3PA spectra (dotted line). Vertical lines mark typical wavelengths used in MPP.

Experiments were conducted using the SZ2080^TM^ prepolymer (Foundation for Research and Technology – Hellas (FORTH)), and photoinitiator 2-benzyl-2-dimethylamino-1-(4-morpholinophenyl)-butanone-1 (IRG369, Sigma Aldrich). The photosensitized mixture was prepared by adding PI (1% w/w of prepolymer) and stirring with a magnetic stirrer until the PI dissolved. Before the experiment, both pure and photosensitized SZ2080^TM^ were kept at 4 °C temperature. Absorbance spectra of the SZ2080^TM^ prepolymer and the IRG369 photoinitiator are shown in Figure 5.

### 2.5 Sample preparation and analysis

Microscope coverslips (REF VBS638, Biosigma, Cona VE, Italy) were cleaned in an ultrasonic bath (EMAG Technologies, Mörfelden-Walldorf, Germany) in isopropanol for 20 min. To strengthen the adhesion between the polymerized objects and the coverslips, they were immersed in a mixture of isopropanol and 3-(trimethoxysilyl)propylmethacrylate (MAPTMS) (40:1 v/v). Afterwards, the coverslips were cleaned with acetone and pure or photosensitized prepolymer was drop-casted onto them. Prepared samples were dried on a hot plate (PZ 28-3T) controlled by programmer (PR 5-3T, Harry Gestigkeit GmbH, Düsseldorf, Germany). When the polymerization experiment was finished, the uncured prepolymer was developed with 4-methyl-2-pentanone. The samples were dried using a critical point dryer (CPD) (K850, Quorum Technologies, East Sussex, UK) and were sputtered with a 10 nm silver layer employing a rotary pumped coater (150R S, Quorum Technologies, East Sussex, UK). A SEM microscope (Prisma E, Thermo Fischer Scientific, Eindhoven, The Netherlands) was used to record images of the obtained suspended lines and determine the polymerization and damage thresholds.

## 3 Results and discussion

**Figure 6:**
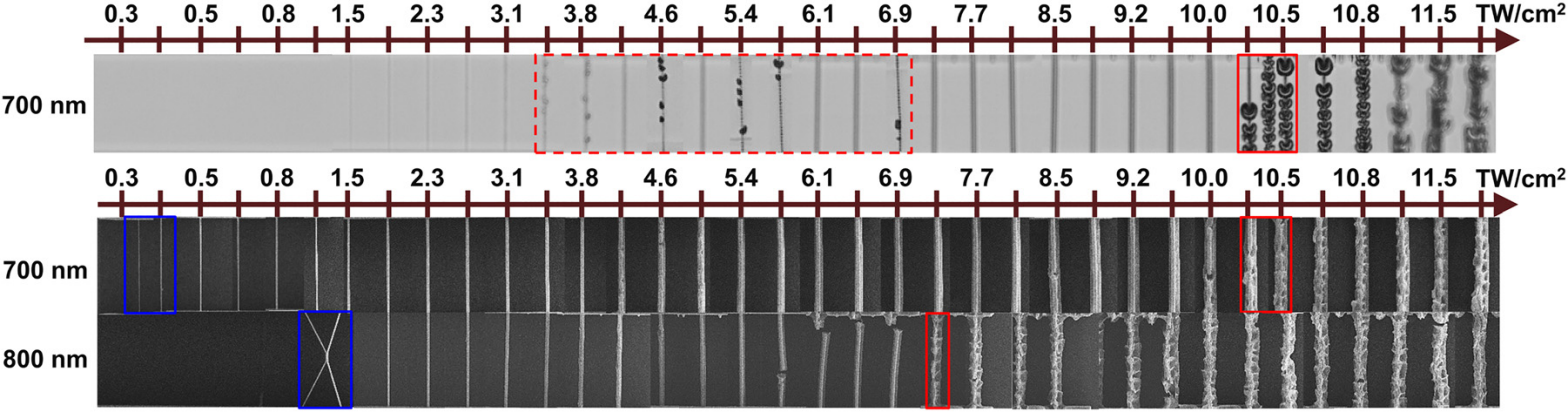
An example of the RBs manufactured using 100 fs pulses at two different *λ* in SZ2080^TM^ + IRG369 prepolymer at different intensities: 700 nm – upper two rows, and 800 nm – bottom row. The first row shows optical images taken during the MPP process. The bottom two rows show SEM images after full development in solvent and CPD. The blue and red zones mark intensities at which the polymerization and optical damage thresholds were observed. The dashed red zone shows an example of bubble formation seen in optical images.

Figure 6 depicts the intensity dependence of the polymerized lines registered in two ways – optically with a CCD camera during the polymerization process and electronically with SEM imaging after the development in solvent and CPD. The RB series were fabricated at a fixed wavelength and pulse duration while varying the intensity level. The intensity required to obtain the first polymerized RB was designated as the polymerization threshold. The value at which the RB started to show observable optical damage was denoted as the damage threshold. As evident from Figure 6, SEM images of fully developed RB samples suggest that the polymerization thresholds at 700 nm and 800 nm with *τ* = 100 fs are 0.35 TW/cm^2^ and 1.03 TW/cm^2^ while the damage thresholds are 10.3 TW/cm^2^ and 7.3 TW/cm^2^, respectively. However, in optical images taken during the polymerization process, the formation of bubbles was observed at lower and non-repetitive intensities, as shown in Figure 6, top row. In this case, the formation of bubbles was observed within the 3.5–7 TW/cm^2^ range, with an average intensity value of 6 TW/cm^2^.

### 3.1 Processing window: from threshold to breakdown

**Figure 7:**
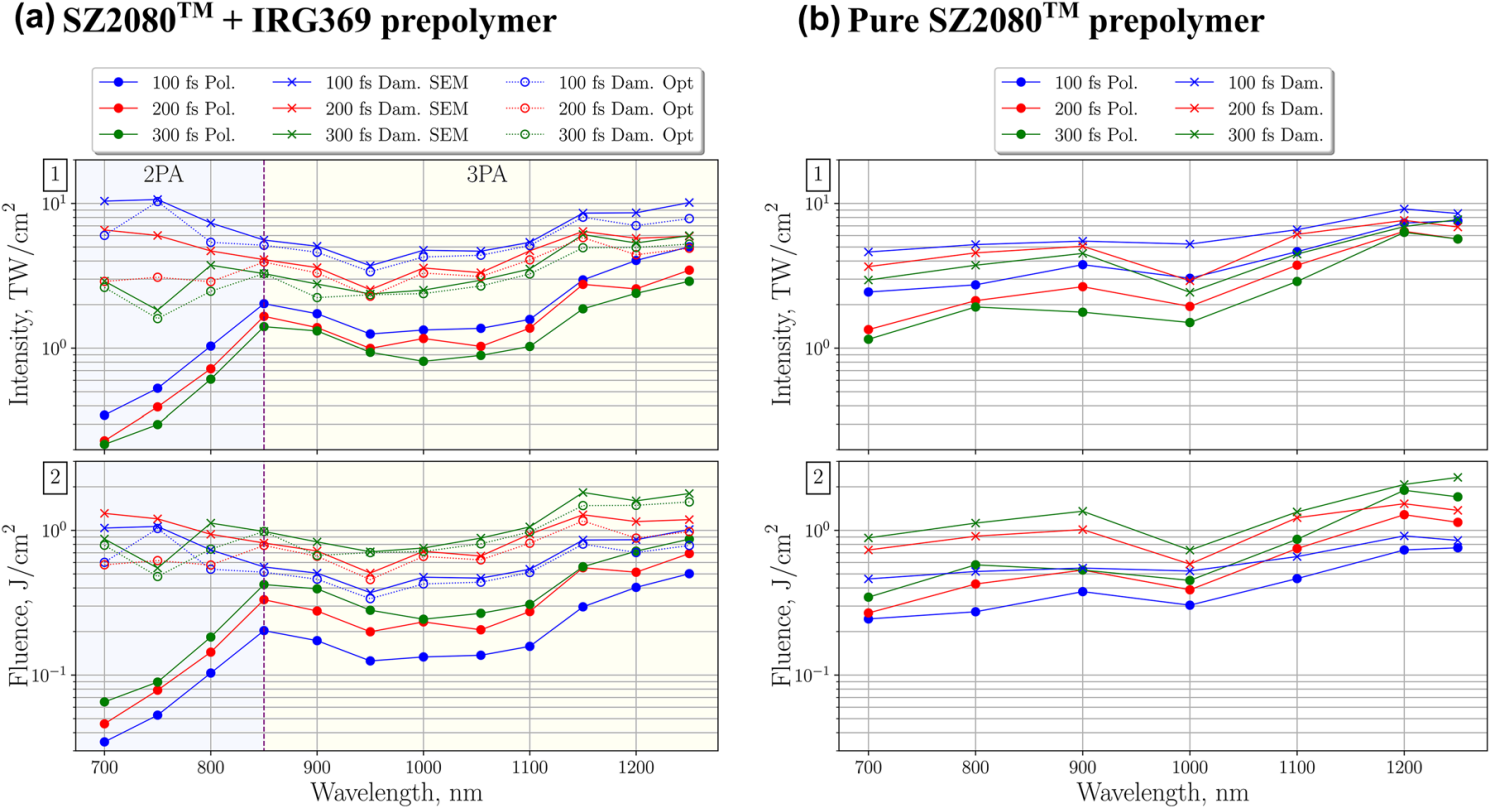
From the top: (1) polymerization threshold (solid lines with solid dot markers) and optical damage threshold (solid lines with cross markers) dependence on *λ*, when *τ* = 100, 200 and 300 fs. SZ2080^TM^ + IRG369 prepolymer includes damage thresholds recorded optically during the MPP process (dashed lines with empty circle markers); (2) fluence level at the same thresholds.

Despite MPP being a thresholded process, it was difficult to identify the polymerization threshold optically due to the nanoscale voxel size and a limited photo-induced refractive index change which is also intensity-dependent [36]. In contrast, the threshold was unambiguous in SEM images, where the first polymerized RB was already structurally strong enough to withstand the development in solvent and CPD. It is noted that the surviving lines were observed using SEM at distinctively lower *I* values in comparison to the optical microscope before the development. The MPP reaction has presumably started at an even lower intensity level; however, the polymerized lines were either discontinuous or structurally weak and collapsed during post-processing. Therefore, it must be taken into consideration that polymerization thresholds determined from SEM images are affected by geometry-related structural strength requirements. Theoretically, the threshold would be lower for shorter RBs or other self-supporting structures. Damage threshold estimates were also different for optical and SEM images. The formation of bubbles was optically seen at a lower intensity level, especially at shorter wavelengths where 2PA was expected (see Figure 7(a)). However, after the development in solvent and CPD, SEM images of the same RB series indicated optical damage threshold at a higher intensity level. This disparity could be explained by solvent post-processing, where the bubbles that were visible optically are washed off the RB surface or partially dissolve due to the large surface area of the bubble. Since post-processing in the solvent is an inseparable part of the MPP process, damage thresholds obtained from SEM images were used in further analysis.

The dependence of the polymerization and optical damage thresholds on the wavelength and pulse duration for both SZ2080^TM^ + IRG369 and pure SZ2080^TM^ prepolymers are summarized in Figure 7. In both prepolymers, MPP was achieved at all wavelengths within the 400–1300 nm range. However, threshold values could not be reliably determined after the objective for sub-700 nm wavelengths due to limited autocorrelator range, thus the data was omitted from the analysis.

The polymerization threshold trend in SZ2080^TM^ + IRG369 prepolymer correlates with the expected multi-photon absorbance spectra (see Figure 5). The lowest polymerization threshold was recorded at 700 nm, which corresponds to the reported 2P absorbance peak in the photoinitiator, centered on 670 nm. This wavelength also corresponds to a considerable level of expected 3PA in SZ2080^TM^. 2PA in the photo-initiator was dominant at the polymerization threshold since the probability for 2P transitions is higher than that for 3P transitions. However, 3PA in SZ2080^TM^ molecules may come into play at higher intensities when most of the photo-initiator molecules have already been transitioned to an excited state. Under such a condition, concentration of the photo-initiator can contribute toward the damage threshold level as will be discussed later. The second polymerization threshold dip was observed at ∼1050 nm, which corresponds to the expected 3P absorbance peak depicted in Figure 5. The transition from 2P to 3P excitation regime was centered on ∼850 nm, while the expected transition in the photoinitiator absorbance spectra is centered on ∼800 nm. The transitional absorption gap was found to be narrower than what the expected absorbance spectra suggests. One possible explanation for this is that prepolymer molecules have complex energy level structure and can store photon excitation energy in a variety of ways including: translational energy, rotational energy, vibrational energy and electronic energy. The combination of these different states leads to a broadening of the spectral absorption lines and can even result in shifted multi-photon absorption peaks.

No clear transition between non-linear absorption regimes could be observed for the optical damage threshold. This is due to optical damage being caused by avalanche ionization rather than just the multi-photon absorption. Optical breakdown thresholds presented in row 1 of Figure 7 show that in 1000–1200 nm range the damage thresholds in SZ2080^TM^ + IRG369 and pure SZ2080^TM^ are nearly identical, with the largest difference of just 20% observed at 1100 nm. This suggests that at the concentrations of photoinitiator used it does not contribute substantially to the damage process. This matches with findings reported by Fischer et al. with several different photoinitiators [37]. In contrast, at shorter wavelengths where 2PA starts to dominate, the addition of IRG369 photoinitiator resulted in up to 2.2× higher optical damage threshold than in pure SZ2080^TM^ prepolymer.

### 3.2 Pulse duration and wavelength dependence of polymerization

The pulse duration was found to affect both polymerization and damage thresholds. As the pulse shortens, the threshold intensity level increases. However, at the same time, the threshold fluence level is reduced, as shown in row 2 of Figure 7. MPP with shorter pulse duration required less optical energy, thus, a reduction in optically-induced thermal effects can also be expected. Cross-linking and radical diffusion are both temperature-dependent processes [38, 39]. In some prepolymers, thermal effects can limit the polymerization resulting in unwanted voxel spread [40], while in others; temperature-accelerated termination helps to achieve smaller voxel size [41]. As shown by the results, tunable *τ* can be used as a variable to control the fluence level, which in turn could provide control over the degree of thermal effects. In general, experimental results show nearly constant inverse *τ*-dependence for the polymerization and damage thresholds in both prepolymers. However, *τ* was found to have a stronger non-linear influence on the damage threshold within the 2PA region in the photosensitized prepolymer. For example, compressing the 700 nm pulse from 300 fs to 100 fs increased the optical damage threshold from 2.9 to 10.3 TW/cm^2^ in the SZ2080^TM^ + IRG369 prepolymer, while a change from 2.9 to 4.6 TW/cm^2^ was observed in pure SZ2080^TM^. This, in turn, contributed toward 2PA region having the widest and *τ*-dependent dynamic fabrication window, which is defined as:

(1) $$\text{DFW}=\frac{I_{\text{dam}}-I_{\text{pol}}}{I_{\text{pol}}}.$$

Here *I*_pol_ and *I*_dam_ are intensity values of the polymerization and damage threshold, respectively.

**Figure 8:**
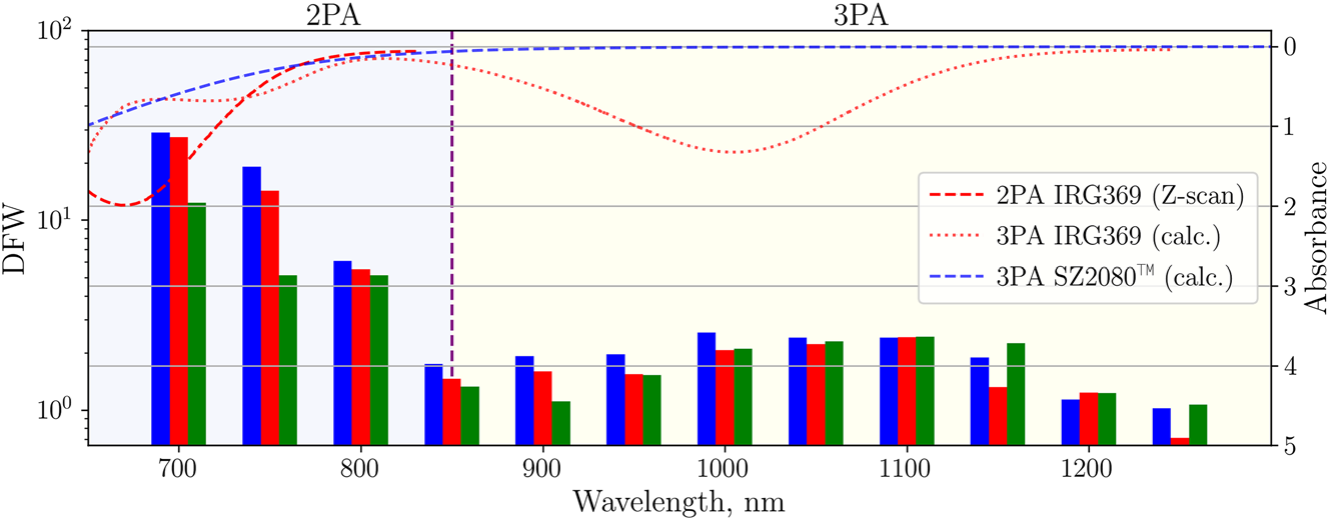
DFW dependence on *λ* and *τ* in SZ2080^TM^ + IRG369 prepolymer. Measured and expected (marked as calc. in the legend) multi-photon absorbance spectra of SZ2080^TM^ and IRG369 are presented on an inverted secondary *Y* axis on the right.

The DFW in SZ2080^TM^ + IRG369 prepolymer is shown in Figure 8 and the values are provided in Table 1. Here we see the transition between 2P and 3P polymerization regimes, which correlates with the expected photoinitiator absorbance spectra from Figure 5 shifted by ∼50 nm. As previously discussed, multi-photon absorbance lines seem to be wider than expected from Figure 5. DFW *λ*-dependence is linked to the fact that the 3P process at longer wavelength requires *I* ^3/2^ higher intensity level than the 2P process, while the optical breakdown threshold increases with wavelength but does not scale directly with the order of the absorption non-linearity. As a result, the 2P polymerization region has higher DFW values than the 3P polymerization region. The highest DFW value of 29 was achieved for 100 fs, 700 nm irradiance. Although 800 nm is a standard wavelength used for MPP, decreasing it by just 100 nm results in the increase of DFW by the factors of 2.4 or even 4.8 for 300 fs and 100 fs pulses, respectively. These findings emphasize the importance of both *λ* and *τ* optimization for maximizing the DFW in 2P polymerization. Regarding 3P polymerization regime, the highest DFW value was recorded at the 1050–1100 nm 3PA peak. Interestingly, the DFW was found to also be *τ*-insensitive in this region.

**Table 1:** DFW dependence on *λ* and *τ* in SZ2080^TM^ + IRG369 prepolymer.

Pulse duration, fs	Wavelength, nm											
	700	750	800	850	900	950	1000	1050	1100	1150	1200	1250
100	29	19.1	6.1	1.8	1.9	2	2.6	2.4	2.4	1.9	1.1	1
200	27.4	14.3	5.5	1.5	1.6	1.6	2.1	2.2	2.4	1.3	1.2	0.7
300	12.4	5.1	5.1	1.3	1.1	1.5	2.1	2.3	2.4	2.3	1.2	1.1

**Figure 9:**
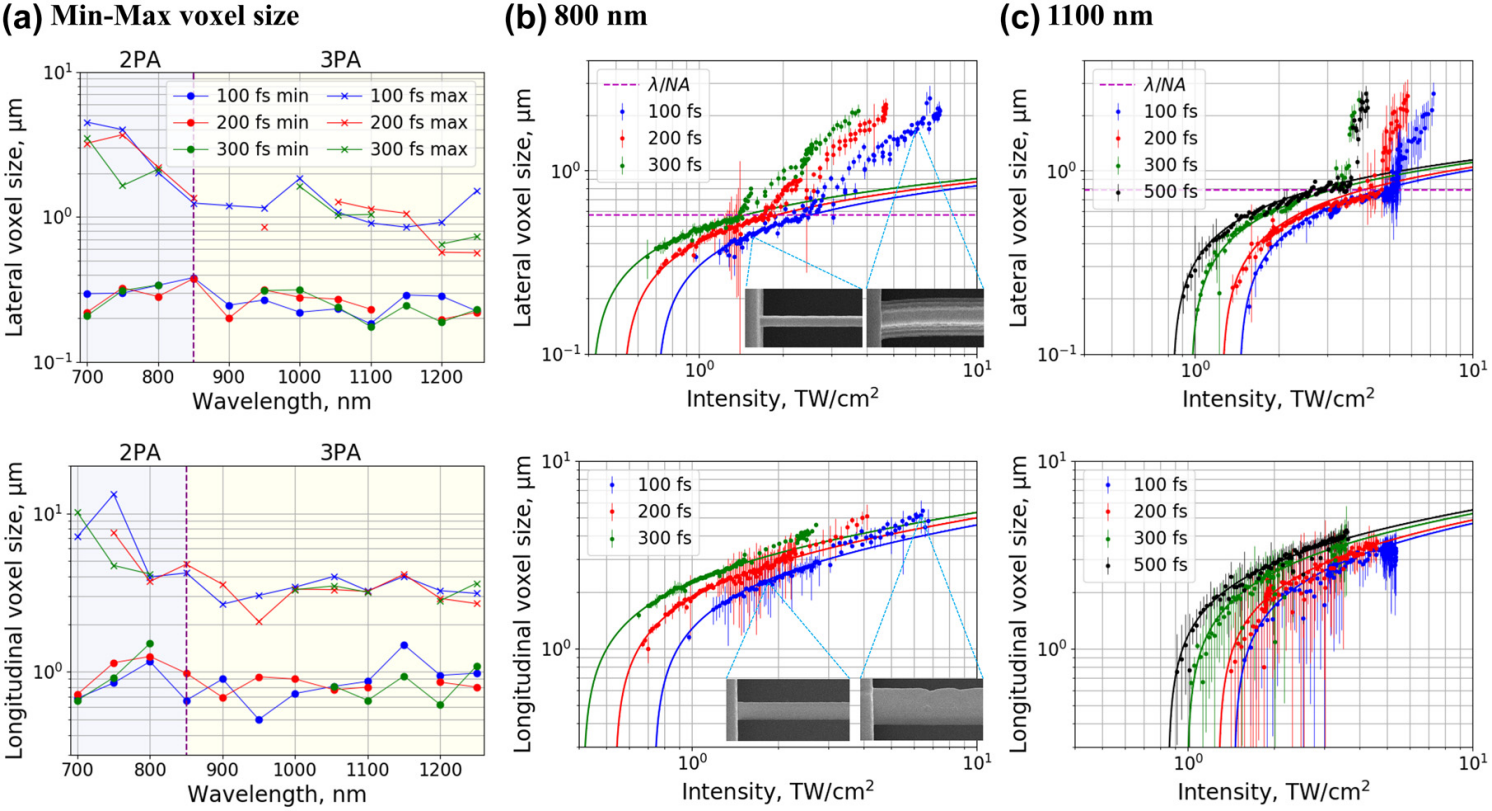
Experimental results of voxel growth. (a) Minimum and maximum voxel size dependence on *λ* and *τ*; (b) and (c) lateral and longitudinal voxel size dependence on the applied intensity with different *τ* for 800 nm and 1100 nm wavelengths respectively in SZ2080^TM^ + IRG369 prepolymer. Solid lines represent voxel growth model curves. Dashed horizontal line marks the diffraction limit. Polarization of the writing beam was set to linear. Sample SEM images of lines polymerized at 1.6 TW/cm^2^ and 6.1 TW/cm^2^ intensity levels are provided for the 800 nm, 100 fs case.

Figure 9 shows the voxel size limits and growth dynamics for the SZ2080^TM^ + IRG369 prepolymer case. The thinnest polymerized lines were obtained at 2PA and 3PA peaks – 0.21 µm and 0.18 µm, respectively, while at other wavelengths, the minimum lateral voxel size was larger. Somewhat surprisingly, thinner lines were obtained at longer wavelengths, despite the concomitant increase in diffraction limit. One explanation for this is higher-order non-linear absorption at longer wavelengths. The maximum obtainable lateral voxel size was found to be strongly *λ*-dependent and generally followed the DFW trend. The widest voxel of 4.5 µm was obtained at 700 nm irradiance. This value dropped to sub-µm levels at wavelengths exceeding that of the 3PA peak. The dependence of the longitudinal voxel size closely correlates with the lateral voxel size. However, longitudinal voxel size was generally significantly greater than the lateral dimension due to beam propagation along this axis. The average aspect ratio obtained at the polymerization threshold varied from 2.9 to 3.7 in 2P polymerization region and from 3.6 to 4 in 3P polymerization region for different pulse duration.

Figure 9(b) and (c) represent the voxel growth dynamics with increasing intensity at several *τ* values when *λ* was set to 800 nm and 1100 nm, respectively. The latter dataset includes additional results obtained with 500 fs pulse duration, which conform to the observed trends. The voxel growth can be approximated using Eqs. (2) and (3) for lateral (*d*_*r*_) and longitudinal (*l*_*z*_) growth, respectively [42].

(2) $$d_{r}=2r_{0}\sqrt{\frac{2}{N}ln\left(\frac{I}{I_{\text{th}}}\right)}$$

(3) $$l_{z}=2z_{r}\sqrt{{\left(\frac{I}{I_{\text{th}}}\right)}^{\frac{1}{N}}-1}$$

Here, *r*_0_ and *z*_*r*_ were fixed parameters for each *λ*, while the order of absorption *N* was variable for each *λ* and the polymerization threshold *I*_th_ was set as a variable with initial experimental value for each *λ* and *τ* combination.

Interestingly, lateral voxel growth was found to depart from the model curve after reaching the size close to the diffraction limit of ∼*λ*/NA. Pulse duration and intensity level had no observable effect on the transition point. After reaching the transitional voxel size, further lateral growth switched to nearly linear *I*-dependence with a slope of *γ* = 1 in the 2PA region and *γ* = 2 in the 3PA region. Similarly, a departure from the expected trend was also observed for longitudinal voxel growth. The transition happened after reaching the longitudinal voxel size of $∼\sqrt{2}z_{r}$ . It must be noted that lateral and longitudinal transition points were correlated and, in the 2PA region, occurred well below the damage threshold. For example, at 800 nm the transitional lateral and longitudinal voxel sizes for a given pulse duration were reached at the same intensity level, equal to ∼1/3 of the damage threshold. However, in the 3PA region, lateral and longitudinal transitions were observed at intensity levels equal to 70–100% of the damage threshold, with the closest equivalence observed at the 3PA peak. At 1100 nm, voxel growth did not diverge from Eq. (3) until the optical breakdown threshold. However, at adjacent wavelengths step-wise transition was observed before optical damage occurred.

The deviation from the expected voxel growth rate could have been caused by the overfilling of the back aperture of the objective. Under such a condition it would behave as a hard aperture causing rings of diffraction orders to form around the diffraction-limited focal point. Once the intensity level for a given pulse duration reached the polymerization threshold in the entire diffraction-limited volume, the outer diffraction rings might have started contributing toward the MPP process, resulting in the step-like change in the power law of polymerization.

### 3.3 Mechanism: step-wise change in power law of polymerization

To obtain further insights into the polymerization mechanisms reflected in the step-like changes of *γ*-slope in the power dependence of the lateral and axial voxel sizes (Figure 9), the energy deposition into the focal volume is considered next. Absorbed energy density per volume [J/m^3^] is driving physical and chemical modifications along the pulse propagation, especially since J/m^3^ ≡ N/m^2^ ≡ Pa. i.e., pressure. This is suggested by the irregular patterns at high-intensity *I*_p_ ≥ 7 TW/cm^2^ in Figure 6. The sole parameter describing the light–matter interaction at the focal region is the instantaneous refractive index $\sqrt{\varepsilon }\equiv \tilde{n}=\left(n+\text{i}\kappa \right)$ , where *ɛ* is the permittivity. The starting photo-polymer is a dielectric-like medium with negligible absorption (1.5 + i0) in the visible and NIR spectral range. The axial length of the polymerized focal region is up to a few micrometers and is much smaller than the pulse length (100 fs corresponds to 30 µm length). It can be thus considered that the entire focal region is exposed to the laser pulse. Upon optical excitation and free carrier generation, the real part of *n* is reduced while the imaginary *κ* is increased; the dielectric breakdown by definition is Re(*ɛ*) = 0 (hence *n* → 0). Both *n* and *κ* contribute to scattering (reflection is a type of back-scattering) and absorption. As revealed by the experiment, the diffraction limit is important for the onset of different polymerization power laws. A logical conjecture is to expect this change to be related to the change in light scattering from the sub-diffraction limited polymerized region since polymerization as densification and crosslinking is usually linked to an increase in *n*. There is a change of phase by *π* at the interface for the transmitted/reflected beam when the refractive index transitions from low to high and the lower *n* value is expected on the optical axis. When the index *n* contrast along the propagation is reversed from high to low, no phase change occurs. A lower reflectance *R* contributes to a stronger absorbance since *A* = 1 − *R*−*T*, where *T* is transmittance. Such redistribution of the pulse energy can enhance local energy deposition and the observed lateral spread of polymerization merging with the Airy disk as discussed earlier.

A stronger absorption due to augmented *κ* via increased free carrier density facilitates a positive feedback loop towards runaway breakdown. However, for short sub-1 ps pulses, this process is clearly controlled in the photo-polymer. Free-carrier absorption, which scales as ∝*λ* ^2^ is a likely contributor to the step-like onset of the lateral voxel increase. This conjecture is probable due to observed stronger presence at longer wavelengths.

Competition between the decrease of *n* and increase in *κ* along the optical axis in a sub-diffraction limited volume can have one counter-intuitive contribution manifested in nanoscale deposition of energy and direct write nanolithography [43]. Namely, the normal component of the E-field (*E*_⊥_) should satisfy the continuity of displacement *ɛE*_⊥_ between the optically excited region on the optical axis and the surrounding pristine resist. Even a small difference in refractive index $\frac{n_{\text{low}}}{n_{\text{high}}}$ translates in intensity enhancement in the depressed index on-axis by a factor of $I_{\text{high}}={\left(\frac{n_{\text{high}}}{n_{\text{low}}}\right)}^{4}I_{\text{low}}$ , since *I* ∝ *E* ^2^ and *ɛ* ∝ *n* ^2^. For example, for *n*_high_ = 1.5 (resist) and 10% index decrease due to photo-excitation and ionization on the optical axis, the intensity is increased by 52%. Larger intensity increase exactly on the optical axis is expected when the material enters the epsilon-near-zero (ENZ) state *ɛ* = (*n* ^2^−*κ* ^2^) + i2*nκ* with *n* ≈ *κ*. With a laser pulse having *ct*_p_ ∼ 100 μm and a focal region of only 1–4 μm, the efficient energy deposition into the optically excited region is expected to take place during the very same pulse. As a result of the near-field on-axis enhancement of the field with a simultaneous increase of absorption (*κ* is augmented due to free carriers), longer axial polymerized features are expected and observed experimentally. The described qualitative picture applies to nanoscale laser writing [43] but not for features that are larger than the diffraction limit. The very same is observed in this experiment in the nanoscale (sub-diffraction) region of polymerization.

There are other factors that can further enhance energy absorption along the optical axis. Due to the tight focusing employed, there is a significant longitudinal E-field component *E*_‖_ ∝ sin *α*, where *α* is the half-angle of the focusing cone. The *E*_‖_ field is not reflected and contributes to a larger energy deposition. This type of energy deposition is referred to as resonant absorption and it has a strong contribution at tight focusing conditions [44]. At increasingly higher irradiance per pulse, close to the dielectric breakdown at ∼20 TW/cm^2^/pulse, the ionized focal volume acts as a metallic nanosphere and E-field enhancements change from the poles to the equator, causing a lateral increase of the E-field strength [45]. This drives a redistribution of the light intensity in the focal volume. Finally, an optomechanical action of high-power pulses *E*_p_/*t*_p_ [W] could take place since Force = Power/*c* and can be accumulated due to a direct absorption (deposition of linear momentum) and reflection (doubled action per photon due to momentum reversal). A 10 nJ pulse of *t*_p_ = 230 fs exerts a force of *F* = 0.15 mN ( $I_{\text{p}}=E_{\text{p}}/{\left(t_{\text{p}}\times \left(\pi 0.61\lambda /\text{NA}\right)\right)}^{2}\approx 9.3$ TW/cm^2^ for *λ* = 500 nm and NA = 1.4). Energy deposition depth is the skin depth at the conditions of exposure *l*_abs_ = *c*/(2*ωκ*) (for intensity *E* ^2^) and can be sub-wavelength at high (pre-breakdown) irradiance.

## 4 Conclusions

MPP in both pure and photosensitized SZ2080^TM^ prepolymers is achievable at all the employed wavelengths and pulse durations without significant difference in minimal line widths (<300 nm). Transitions between the orders of non-linearity (2PA to 3PA) are observed via changes in the polymerization threshold as the wavelength is varied (≈at 850 nm). Damage thresholds are comparable whether PI is used or not at longer wavelengths (>900 nm) but surprisingly are up to 2.2× lower for pure material at shorter wavelengths (<800 nm). Thus, the presence of the PI widens the DFW both by increasing of the damage threshold and reducing the polymerization threshold. In the photosensitized material, the DFW varies significantly for different wavelengths (from 1 at 1250 nm to 29 at 700 nm). Although both the polymerization and optical damage thresholds increase when pulse duration is reduced from 300 fs to 100 fs, the DFW maintain increment as well, especially at shorter wavelengths. The lowest fluence level required for polymerization was observed at pulse duration of 100 fs suggesting that GDD control can be used as a tool to indirectly control the rate of thermal effects occurring during MPP. The observed lateral and longitudinal voxel growth dynamics reveal a step-like change in the intensity dependence of polymerization. This result can be interpreted as a consequence of the change in the instantaneous refractive index: as material enters the epsilon-near-zero state, a larger portion of the incident light energy is absorbed. The polymerization threshold differs significantly when obtain from using optical microscopy or SEM, the latter revealing significantly more surviving lines produced with lower *I* after the development.

The systematic findings of this study uncover the complexity of MPP mechanisms and emphasize the importance of wavelength and pulse duration control for the optimization of the femtosecond laser 3D nanolithography. Having in mind that there are plenty of monomers and photo-initiators that are used to make photoresists for MPP, a tunable-wavelength laser system with pulse GDD control becomes a useful tool for the investigation of polymerization thresholds. A deeper understanding of the underlying MPP mechanisms is important for increasing the DFW and enabling polymerization of novel cross-linkable materials.

## Supplementary Material

Supplementary Material Details
