# Supplementary material for: 3D nanopolymerization and damage threshold dependence on laser wavelength and pulse duration
Source: Nanophotonics. 2023 Jan 13;12(8):1537–48. doi: 10.1515/nanoph-2022-0629 (PMC11502003; doi:10.1515/nanoph-2022-0629)
Supplement: Supplementary file 1 — Supplementary Material Details [file j_nanoph-2022-0629_suppl.docx]

Danielius Samsonas, Edvinas Skliutas, Arūnas Čiburys, Lukas Kontenis, Darius Gailevičius,
Jonas Berzinš, Donatas Narbutis, Vytautas Jukna, Mikas Vengris, Saulius Juodkazis, and
Mangirdas Malinauskas

**Supplementary information for the manuscript** “*3D nanopolymerization and damage threshold dependence on laser wavelength and pulse duration”*

Transmittance of the objective lens (Zeiss Plan-Apochromat 100× 1.4 NA item number 420790-9900-000) was measured to evaluate the light intensity at the sample. The measurement was performed using a broadly wavelength-tunable laser source CRONUS-3P (Light Conversion), motorized XY scanning stage 8MTF-75LS05 (Standa) for objective positioning in and out of the beam path, PDA10A (Thorlabs) Si fixed gain detectors for 𝜆 ≤ 1100 nm and PDA30G-EC (Thorlabs) PbS amplified detectors for 𝜆 > 1100 nm. A round wedge prism was used to split the beam into two parts. One part was guided to the reference detector and the other to the detector after the objective. Transmittance was calculated by dividing the signal obtained with the objective in the beam path by the signal obtained without the objective. Reference signal was used to account for energy fluctuations from pulse to pulse. Immersion oil was not used during the measurement. Figure S1 depicts measured transmittance and the data provided in manufacturer’s datasheet available at [tps://www.micro-shop.zeiss.com/en/us/shop/objectives/420790-9900-000/Objective-Plan Apochromat-100x-1.40-Oil-M27](https://www.micro-shop.zeiss.com/en/us/shop/objectives/420790-9900-000/Objective-Plan%20Apochromat-100x-1.40-Oil-M27). Measured transmittance was found to be lower that stated in the datasheet for 𝜆 = 400–750 nm, while it conformed well with the manufacturer’s data for 𝜆 = 800–1150 nm. Additional measured data points for 𝜆 = 1200–1300 nm showed a decreasing tendency in the transmittance. In the main text intensity at the sample was calculated using the measured transmittance.


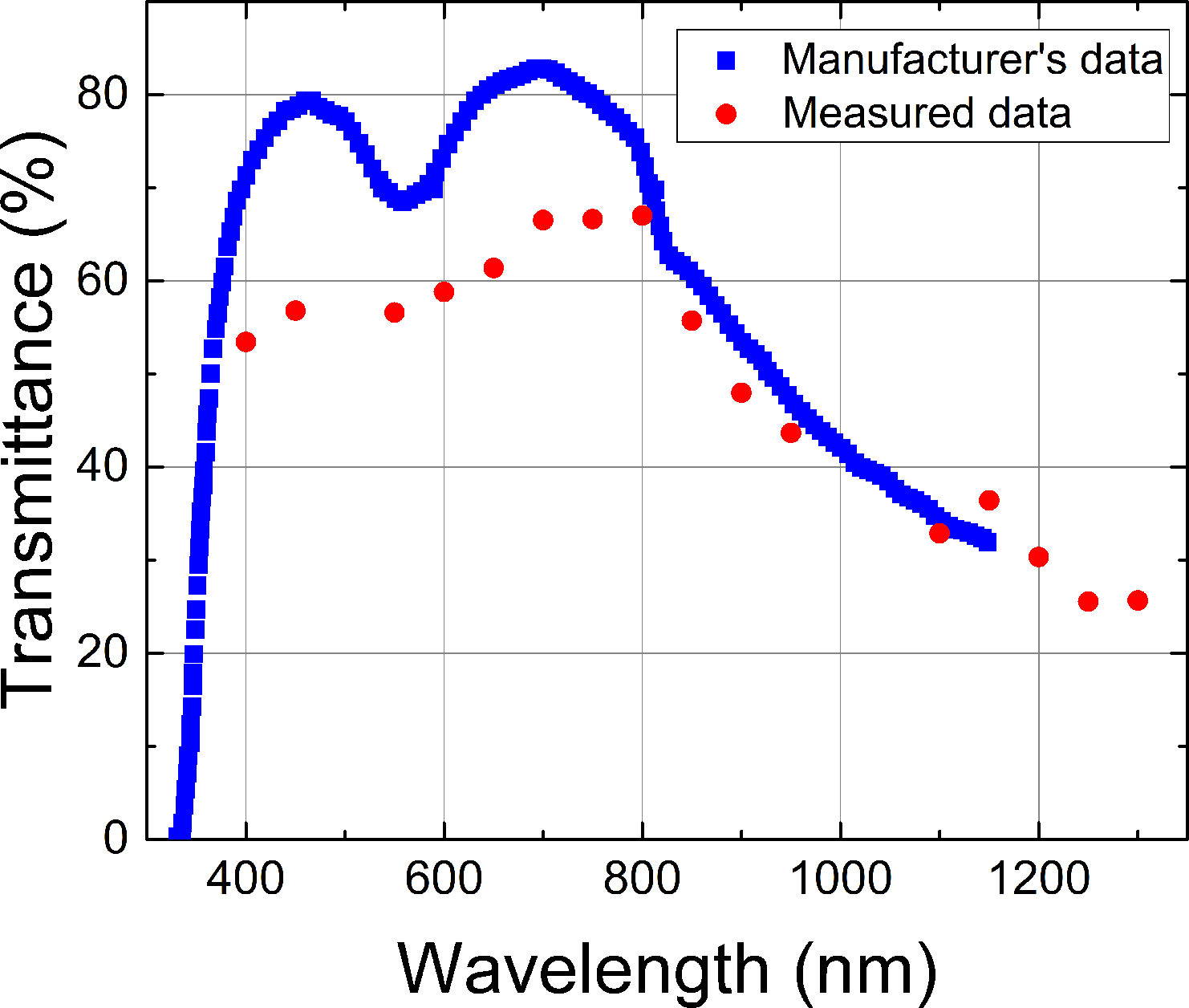


**Fig. S1. Transmittance curve of the Zeiss Plan-Apochromat 100× 1.4 NA objective. Blue dots represent data provided by the manufacturer, red ones – measured by article authors.**
